# Combined approach with negative pressure wound therapy and dermal substitute for extravasation injury: Why can't they be friends?

**DOI:** 10.1016/j.jpra.2020.06.004

**Published:** 2020-06-27

**Authors:** M. Faenza, G.A. Ferraro, F.P. Fonzone Caccese, P. Verolino, S. Papale, P. Di Costanzo, G. Pieretti, S. Izzo, A. Fonzone Caccese

**Affiliations:** a Università degli Studi della Campania Luigi Vanvitelli, Multidisciplinary Department of Medical Surgical and Dental Specialties, Plastic Surgery Unit, Piazza Luigi Miraglia, 2, 80138 Naples, Italy; b Azienda Ospedaliera Universitaria Policlinico, Plastic Surgery Unit, Piazza Luigi Miraglia, 2, 80138 Naples, Italy; c Università degli Studi della Campania Luigi Vanvitelli, Ph.D. Training Program in Medical, Clinical and Experimental Sciences.- Piazza Luigi Miraglia, 2, 80138 Naples, Italy; d Ospedale dei Pellegrini, Hand and Peripheral Nerves Surgery Unit, Via Portamedina alla Pignasecca, 41, 80134 Naples, Italy

## Introduction

Extravasation is defined as an accidental escape of chemotherapy drugs from administration sites, both intravenous and intra-arterial, into the subcutaneous and subdermal tissues.

**Fig. 1:**
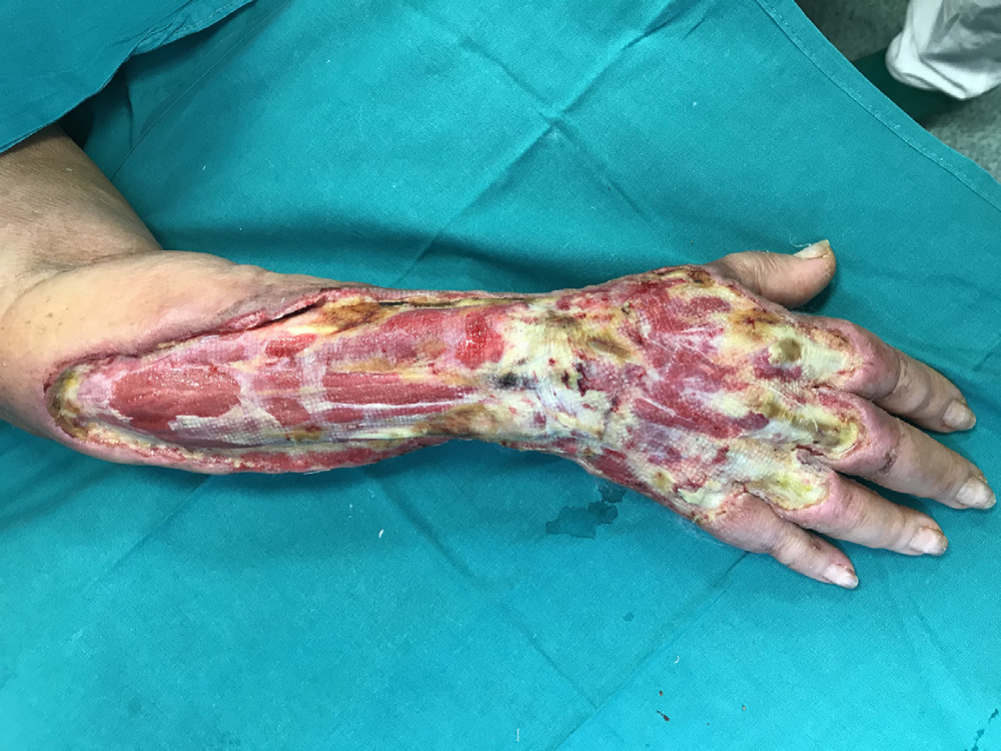
clinical aspect of the wound before surgical debridement.

**Fig. 2:**
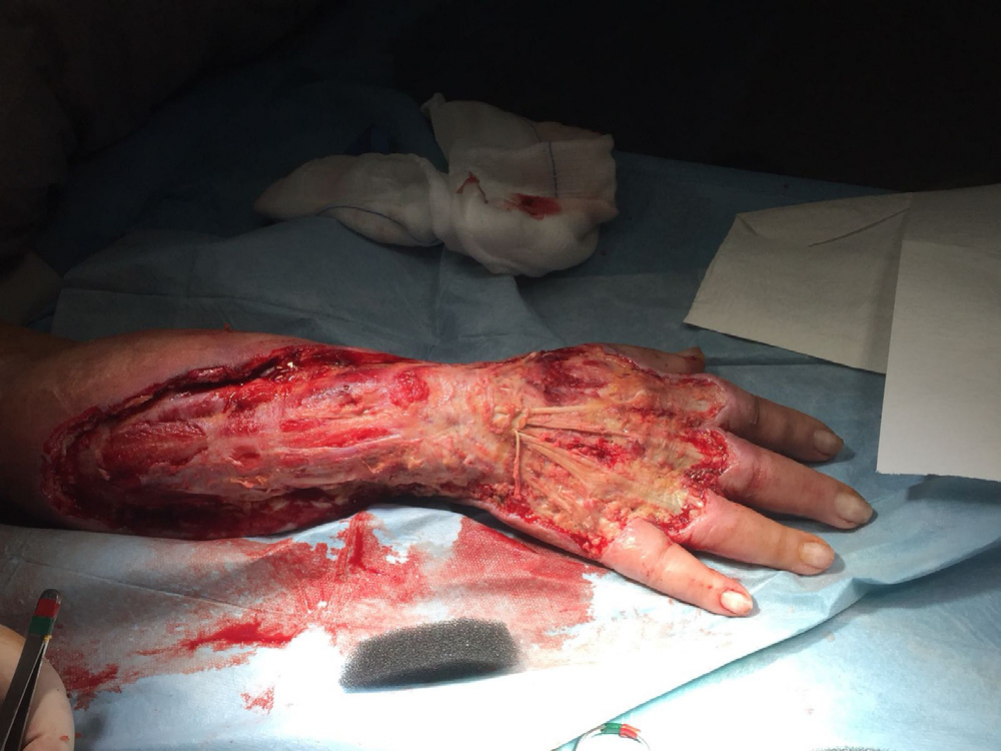
clinical aspect of the right forearm and dorsum of the hand after radical necrectomy with exposure of tendons and muscle.

**Fig. 3:**
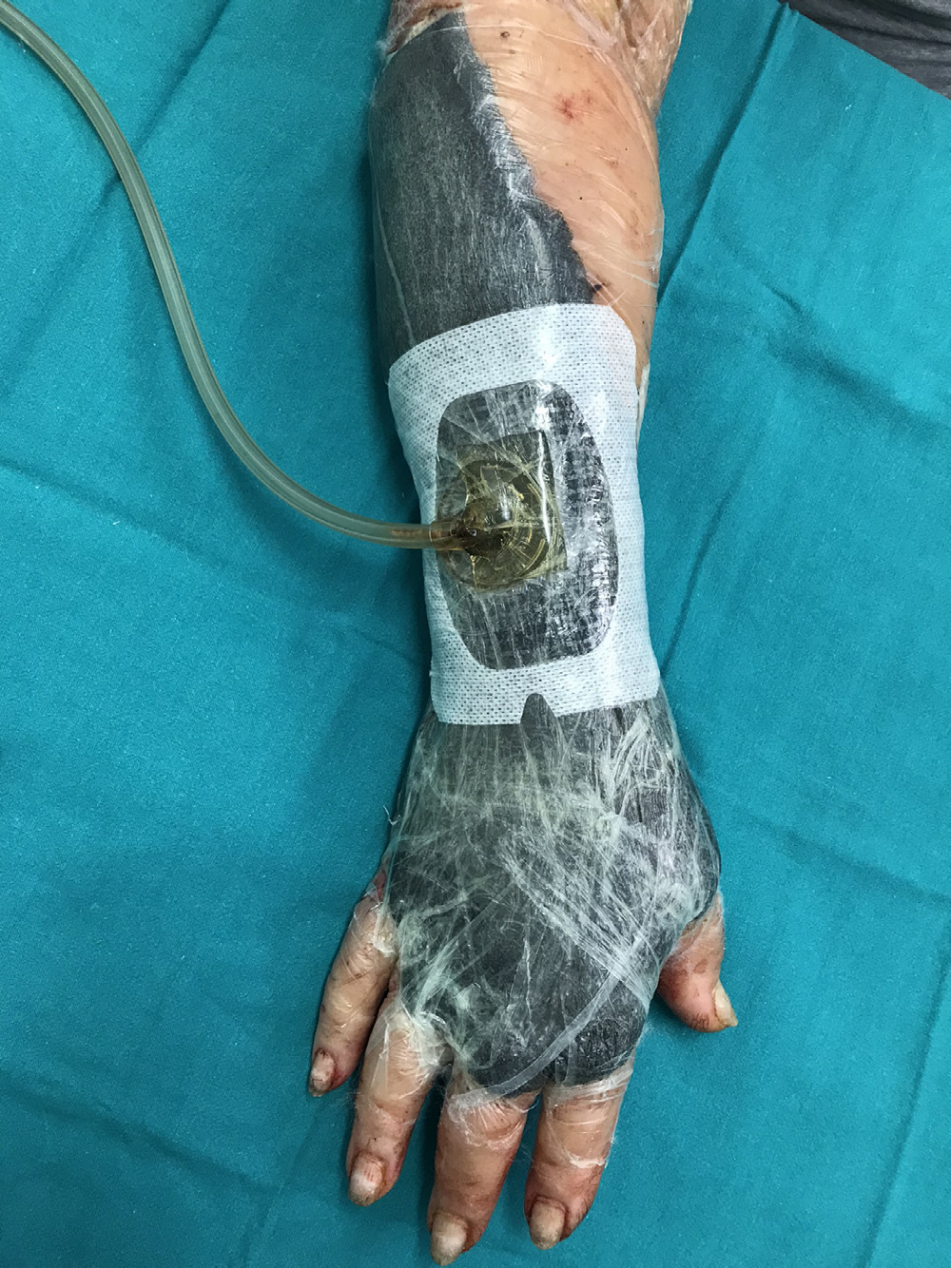
application of Negative Pressure Wound Therapy over the dermal substitute.

**Fig. 4:**
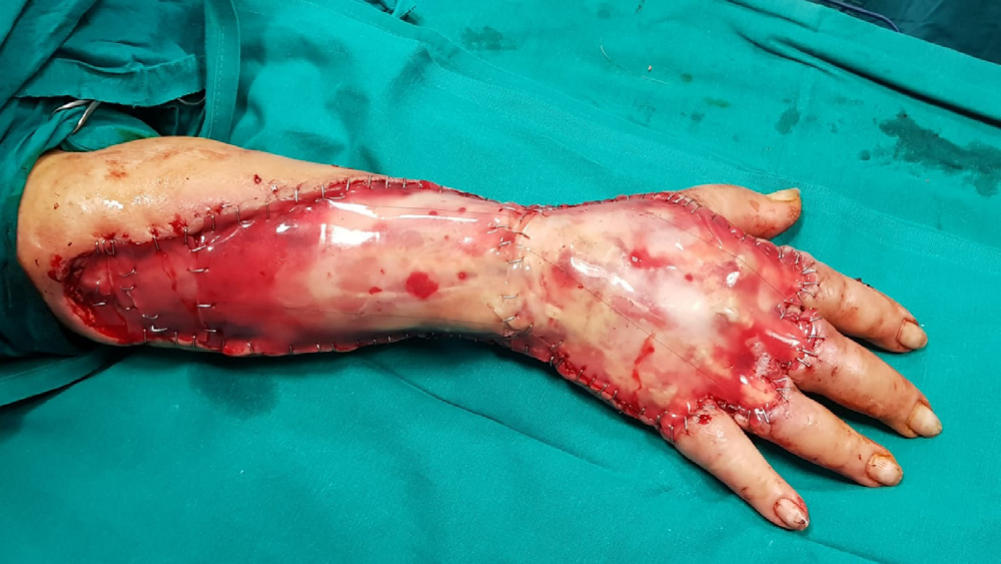
clinical aspect of the dermal substitute completely incorporated after three weeks of NPWT application, notice the multiple stab incisions we made on its surface in order to facilitate fluid and residual drug drainage.

**Fig. 5:**
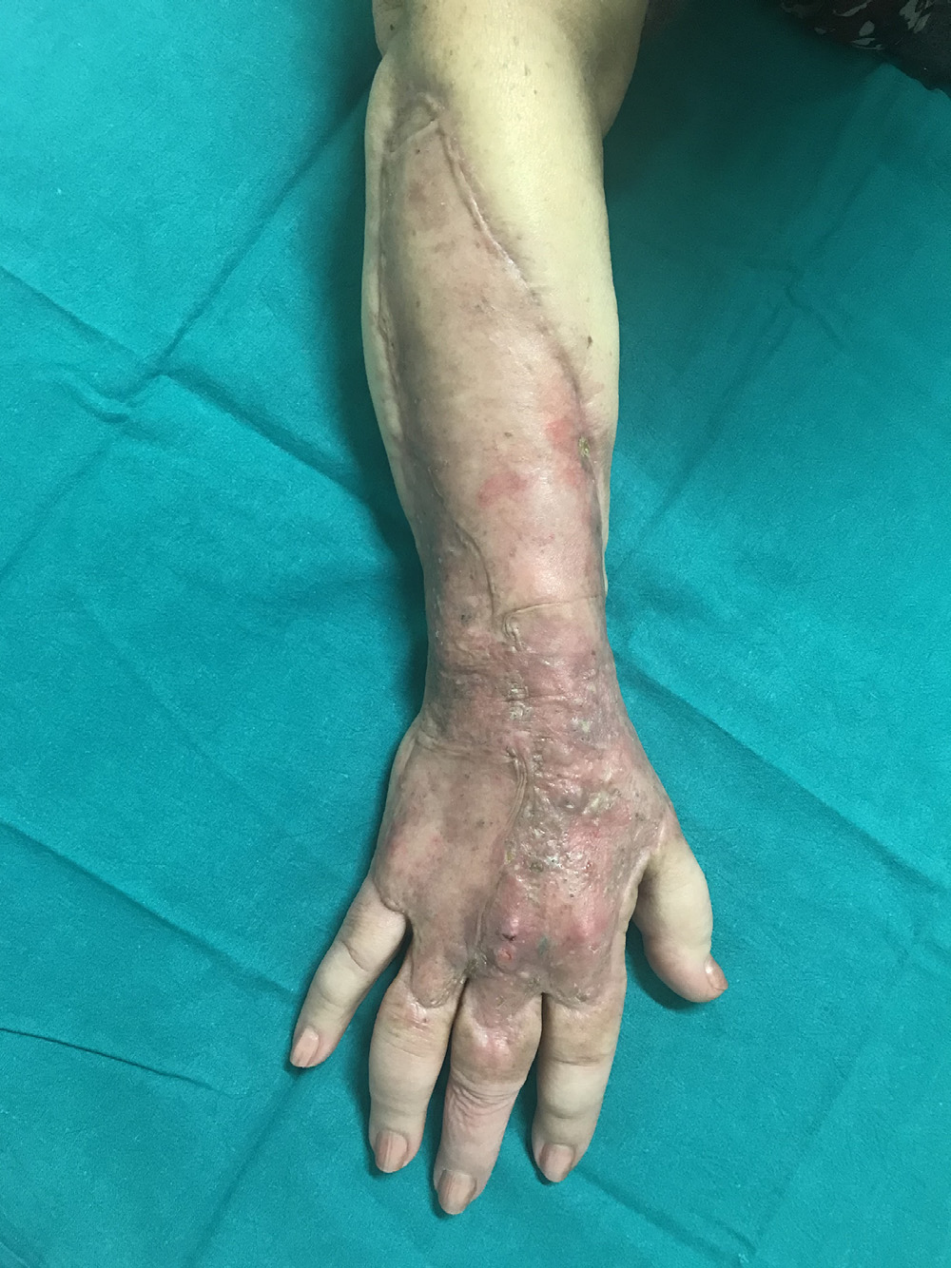
clinical aspect of complete healing at 6 weeks after the radical debridement.

According to the literature, incidence rates of extravasation are estimated between 0.01% and 7%.**^1^****^,^****^2^**

Chemotherapy drugs are classified into three main categories according to their capability of promoting tissue damage in the case of accidental extravasation: irritants, vesicants and non-vesicants.

Clinical signs are various and range from mild erythema to painful necrosis, ulceration and damage of deep anatomical structures. The extent of damage is strictly based on concentration, volume and nature of the extravasated drug.

Epirubicin is an anthracycline agent used in the treatment of several oncologic diseases such as breast, gastric and esophageal cancers. In terms of ability to cause local damage epirubicin is classified as a vesicant agent that acts binding some DNA compounds. Anthracyclines remain in tissues for months following an extravasation injury, promoting damage to surrounding tissues leading to a full-thickness necrosis, that is the reason why early diagnosis is crucial.

Extravasation must be suspected if any of the specific signs or symptoms, such as tingling, burning, swelling, pain and redness, are present at the injection site. Once the extravasated agent has been identified and classified, neutralization or dilution can be started. The aim of the early management is the demarcation of the area of damage in order to monitor its evolution.

Conservative treatment is preferable for most extravasations as it can be difficult to predict how they will heal; however early surgical excision may be favored with more harmful chemical agents.

Unfortunately, extravasation injuries are often underestimated, unrecognized, untreated and probably not adequately reported, consequently these patients are referred to medical and surgical consultation with signs of advanced tissue damage.

In the above-mentioned cases, radical necrectomy and subsequent reconstruction are mandatory.

## Case report

A 68-years-old female patient with a history of breast cancer, previously treated by left radical mastectomy and axillary dissection, was referred to our unit with epirubicin extravasation causing an extensive necrosis of the skin and subcutaneous tissue of the dorsal aspect of the right forearm and dorsum of the hand, which occurred 3 weeks before in another hospital, where it was conservatively treated with calcium alginate topical dressings.

At the time of referral to our unit the area of damage was coarsely demarcated, but the wound bed was still partially covered with fibrin and non-vital tissues. For that reason a surgical radical debridement was performed with complete exposure of muscle and tendons and negative pressure wound therapy (NPWT) positioning.

The first dressing change was done after 48 h in order to check the viability of the wound bed, which appeared to be well vascularized and without signs of infection.

After 3 days, Integra^Ⓡ^ double layer was applied on the tissue defect and sutured with metal clips, we also made several stab incisions on the dermal substitute in order to facilitate residual fluid drainage. The wound coverage was performed with V.A.C. Therapy^Ⓡ^ set to −75 mmHg in continuous mode. Dressing changes were carried out every 4 days and, after 3 weeks, the external silicon layer was removed. The integration of the dermal substitute was checked and we covered the wound with split-thickness skin grafts. Instead of the traditional bandage, we applied V.A.C. Therapy^Ⓡ^ on the grafted area using the same above-mentioned negative pressure regimen.

Skin-graft take was between 90% and 95% with complete healing of the wound within 15 days.

Six weeks after the first surgical debridement, the patient could resume chemotherapy administration Fig. 1, Fig. 2, Fig. 3, Fig. 4 and 5.

## Discussion

According to the literature, there are a variety of strategies for treating extravasation injuries, such as stab and squeeze, liposuction, surgical debridement, and NPWT.

Despite the amount of procedures, the ideal treatment has to be fast and effective in reducing the concentration of the extravasated drugs in order to stop the progression of the damage.

Once the situation has been stabilized, the time of reconstructive surgery comes and generally follows the principles of the classic reconstructive ladder: from direct closure to free flaps.**^3^****^,^****^4^**

In 2011, Onesti et al.**^5^** presented a case series of extravasation injuries treated with dermal substitutes, demonstrating how these devices are useful in increasing the healing rate and reducing economic aspects.

In 2014, Tiwari**^6^** conducted an experimental study on two groups of rabbits with extravasation necrosis established by intradermal doxorubicin injection, one group of animals was treated with the conventional dressing, whereas NPWT was applied to the other group. This study demonstrated that the reduction of wound areas at the end of the treatment was smaller in the NPWT group.

The employment of NPWT systems in a variety of wounds have shown many advantages: improved angiogenesis and granulation tissue formation, accelerated wound closure, and reduced infection rates.**^7^** In addition, due to the above-mentioned properties, NPWT can also be used as an intermediate step in wound bed preparation for delayed reconstruction procedures.**^8^**

Vascular ingrowth is fundamental, both in skin grafts and in dermal template incorporation, for that reason many papers have suggested how NPWT supports and speeds up these processes via evacuating fluid, decreasing edema, immobilizing the grafts, and increasing blood perfusion.**^9^**

Recently Hutchinson et al.**^10^** and Attia et al.**^11^** presented a case series in which they demonstrated how dermal templates combined with NPWT can be safely employed to treat complicated wounds.

In this paper we report an extensive epirubicin extravasation injury of the upper limb successfully treated with surgical debridement and coverage with dermal substitute and NPWT as a first step procedure. The rationale of our therapeutic plan was to combine the removal of potential residual extravasated drug also after the radical debridement and favor dermal matrix incorporation over exposed tendons and muscles. This combined approach in these clinical situations can increase the speed of the whole healing process, which is highly desirable in these cancer patients that are inherently at high risk for several causes and whose main goal is to resume chemotherapy administration as soon as possible.
